# X-ray spectroscopy characterization of self-assembled monolayers of nitrile-substituted oligo(phenylene ethynylene)s with variable chain length

**DOI:** 10.3762/bjnano.3.2

**Published:** 2012-01-05

**Authors:** Hicham Hamoudi, Ping Kao, Alexei Nefedov, David L Allara, Michael Zharnikov

**Affiliations:** 1 Angewandte Physikalische Chemie, Universität Heidelberg, D-69120 Heidelberg, Germany; 2 Departments of Chemistry and Material Science, Pennsylvania State University, University Park, PA16802, USA; 3 Institut für Funktionelle Grenzflächen, Karlsruher Institut für Technologie, D-76344 Eggenstein-Leopoldshafen, Germany

**Keywords:** nitrile substitution, oligo(phenylene ethynylene), self-assembled monolayers, twist angle, X-ray absorption spectroscopy

## Abstract

Self-assembled monolayers (SAMs) of nitrile-substituted oligo(phenylene ethynylene) thiols (NC-OPE*n*) with a variable chain length *n* (*n* ranging from one to three structural units) on Au(111) were studied by synchrotron-based high-resolution X-ray photoelectron spectroscopy and near-edge absorption fine-structure spectroscopy. The experimental data suggest that the NC-OPE*n* molecules form well-defined SAMs on Au(111), with all the molecules bound to the substrate through the gold–thiolate anchor and the nitrile tail groups located at the SAM–ambient interface. The packing density in these SAMs was found to be close to that of alkanethiolate monolayers on Au(111), independent of the chain length. Similar behavior was found for the molecular inclination, with an average tilt angle of ~33–36° for all the target systems. In contrast, the average twist of the OPE*n* backbone (planar conformation) was found to depend on the molecular length, being close to 45° for the films comprising the short OPE chains and ~53.5° for the long chains. Analysis of the data suggests that the attachment of the nitrile moiety, which served as a spectroscopic marker group, to the OPE*n* backbone did not significantly affect the molecular orientation in the SAMs.

## Introduction

Current semiconductor microelectronics devices, although very efficient and compact, are being pushed to their physical limits in terms of further miniaturization with associated issues such as electrical leakage and heat dissipation, and hence this is driving consideration of entirely new types of platforms. One particular idea being actively investigated is molecular electronics, which involves the use of organic molecules as potential circuit elements or components, such as conductors, rectifiers, transistors, and logic gates [1–2]. An important structural element of all such device molecules is an electrically functional molecular unit, which in the simplest case is represented by a conducting oligomeric molecular chain, often termed a “molecular wire”. The charge transport properties of this chain are an essential factor affecting the performance of the entire molecular device. In this context, transport properties of several potential molecular wires, including alkyl, oligophenyl, and oligo(phenylene ethynylene) (OPE) chains have been studied by a variety of different techniques including, for example, conducting-probe mercury drops [3–5], break junctions [6–11], scanning-microscopy tips [12–18], in-wire junctions [9], and cross-nanowire junctions [19]. For most of these measurements the molecular wires were assembled on a conductive substrate, serving as the bottom electrode, by using self-assembled monolayer (SAM) methods. For this purpose, oligomeric chains were combined with a suitable anchor (head) group having a strong affinity to the selected substrate. The most frequently used group in this regard is thiol, which allows SAM-like assembly of the molecules on coinage metal and various semiconductor substrates, for example Au and GaAs, respectively. Another essential element of the experiments is the variation of the length of the molecular wire [3–4,12,14,18,20], which allows further insight into the mechanism of conductance, described as nonresonant superexchange tunneling in most cases [21], and gives the capability to determine essential characteristic parameters, most importantly the attenuation factor describing the trend of exponential tunnelling current versus molecular length. The interpretations of these types of results depend crucially on the actual physical and structural characteristics of the molecules in the SAMs, for example, packing density, molecular orientations, and molecular conformations; and yet in many cases these characteristics are neither precisely controlled nor measured, but simply assumed to be similar to those of other types of molecules and that they do not vary with different lengths of oligomers in the same series.

Considering the variety of electrically functional molecules of interest in molecular electronics, the class of molecules based on simple oligomers of phenylene–ethynylene units is of particular importance for several reasons. First, the OPE chain is one of the most effective conductors among the available molecular wires [22–23]. Second, the electrical properties of the OPE derivatives can be varied significantly by relatively minor chemical modifications [1,13,17,24–25]. In particular, a nonfunctionalized OPE-type molecule behaves as a molecular rectifier [23], whereas, when functionalized in specific ways with nitro, amino or fluoro groups, negative differential resistance can be observed [26–28]. Finally, the electrical properties of OPE-based molecules have been reported to be affected by the local environment, which makes the issue of molecular packing especially significant [9]. For these reasons OPE types of molecules are ideal for fundamental studies.

**Figure 1:**
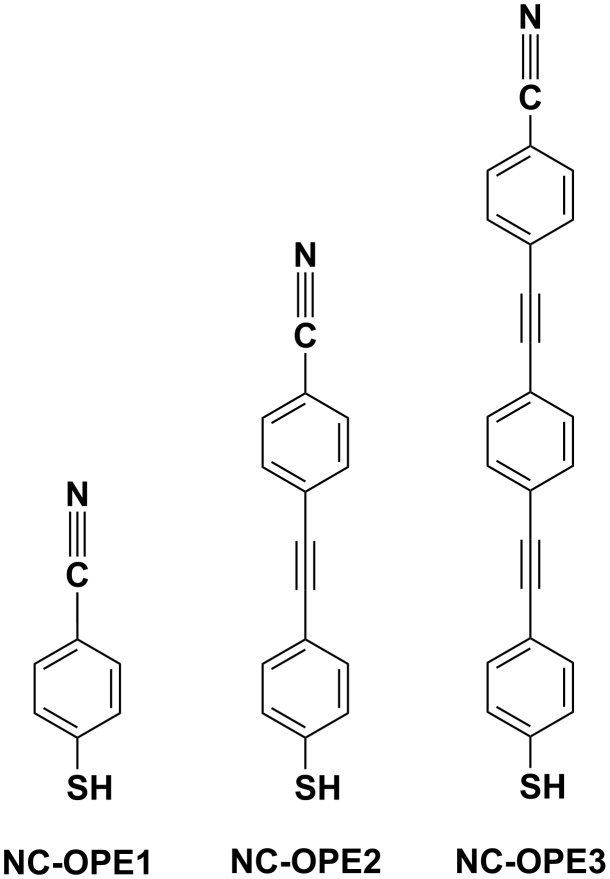
A schematic drawing of the target molecules along with their acronyms.

In this context, we present here the results of the detailed spectroscopic characterization of a series of nitrile-substituted thiolated OPEs assembled as SAMs on Au(111). A schematic drawing of the molecules in this study is presented in Figure 1 along with their acronyms; these molecules are nitrile-substituted thiophenol (NC-OPE1), nitrile-substituted tolanethiol (NC-OPE2), and nitrile-substituted 4-[4′-(phenylethynyl)phenylethynyl]benzenethiol (NC-OPE3). As seen in Figure 1, the length of the OPE chain was varied from one to three structural units, which is the typical length range of the transport experiments. The nitrile tail group served as a spectroscopic marker for X-ray measurements (see below), which allowed the use of electronic excitations to probe directly both the molecular tilt and twist [29–30]. In addition, this moiety can serve as a specific group that can be resonantly excited by X-rays to leave an excited electron on the CN group whose decay by charge transfer (CT) to the substrate can be followed to provide CT lifetimes through the molecular wires [31–33].

The SAM structures of the NC-OPE types of molecules have not been addressed previously (except for a resonance Auger spectroscopy study [33]), although some results on the structure and molecular packing in the SAMs of nonsubstituted OPE have been reported. In particular, based on STM data, Dhirani et al. reported that the degree of order in OPE SAMs on Au(111) increases with chain length. The SAM of the simple molecule thiophenol (OPE1) exhibited no periodicity, that of tolanethiol (OPE2) showed a certain (although poor) degree of order, and that of 4-[4′-(phenylethynyl)phenylethynyl]benzenethiol (OPE3) displayed a highly ordered pattern, which was consistent with a 2√3×√3 structure [20]. These results were supported by further STM [34–35] and AFM [36] studies, which reported no ordered structure in OPE2/Au [34] and a high structural order in OPE3/Au [35–36]. However, in contrast to [20], a noncommensurate structure with a rectangular unit cell was observed for OPE3/Au in [35], while a basic √3×√3 arrangement was recorded in [36]. Whereas the reasons for the above discrepancies are not clear yet, the molecular packing densities in all three STM/AFM studies [20,35–36] were quite similar and close to those of alkanethiolate (AT) SAMs on Au(111). Furthermore, in addition to the STM/AFM characterization, molecular organization in OPE3/Au was probed by infrared-reflection spectroscopy (IRS) [36] and near-edge X-ray absorption fine-structure (NEXAFS) spectroscopy [37]. The average tilt angle of the OPE3 backbone was estimated at 33 ± 18° in [36] and 30 ± 5° in [37], while the twist angle of the backbone with respect to the tilt plane was estimated at 31 ± 6° in [36]. Finally, the preparation of well defined, nonsubstituted and F-, CH_3_-, CF_3_-, and OCH_3_-substituted OPE SAMs on gold with variable length *n* of the OPE chain (*n* ranging from one to three structural units) was described in [38]. The authors, however, presented only results for the SAM-induced work-function tuning and did not provide any information about the SAM structure or packing density.

## Results

### High-resolution X-ray photoelectron spectroscopy

**Figure 2:**
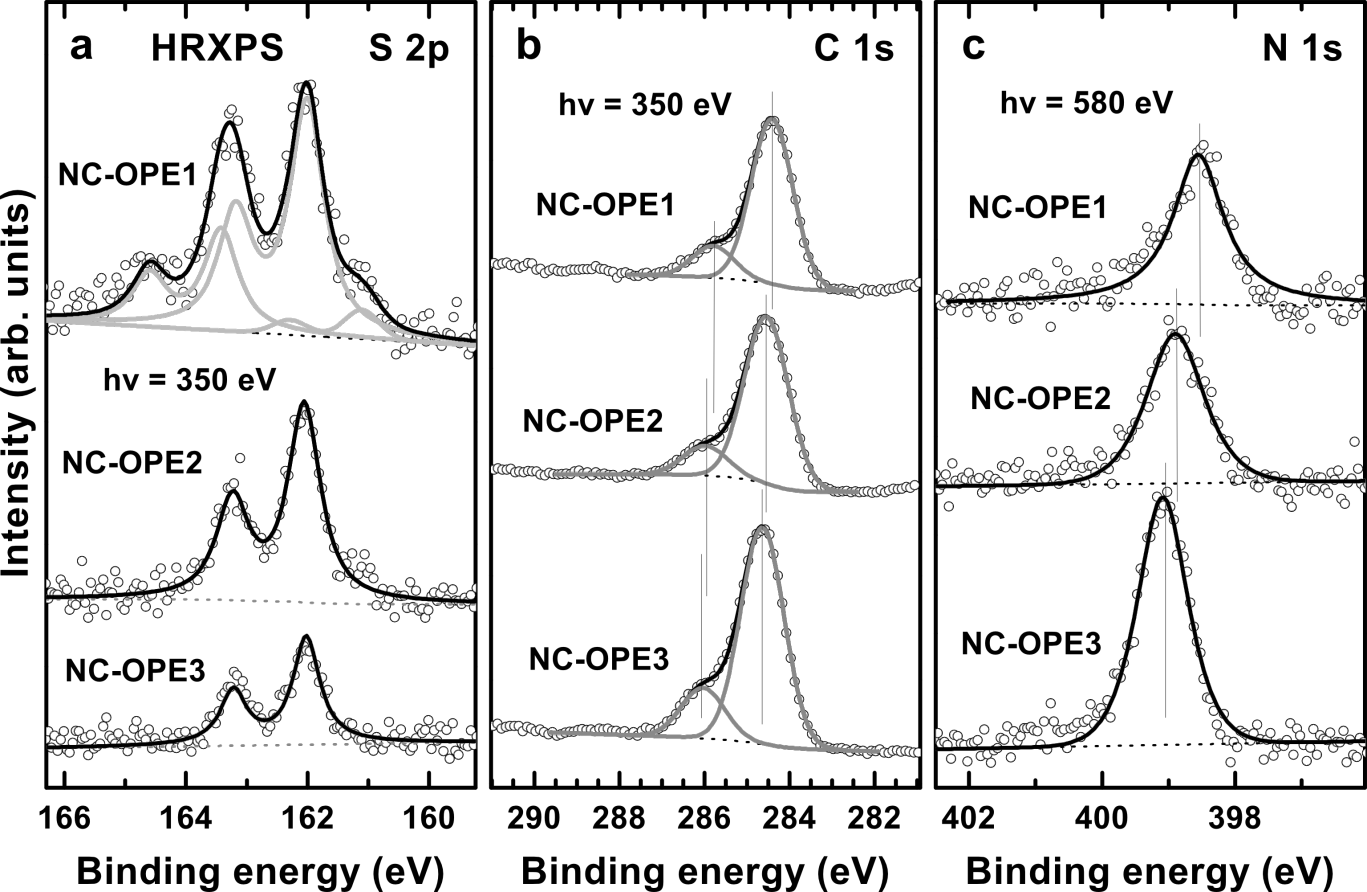
S 2p (a), C 1s (b), and N 1s (c) HRXPS spectra of the target SAMs acquired at photon energies of 350 eV (S 2p and C 1s) and 580 eV (N 1s). Some spectra are decomposed into the individual contributions related to the different species; see text for details. Vertical solid lines mark the positions of the individual emissions in (b) and (c).

High-resolution X-ray photoelectron spectroscopy (HRXPS) provides information about the identity, character, integrity, chemical composition, and effective thickness of the target films. The S 2p, C 1s, and N 1s HRXPS spectra of the target SAMs acquired at photon energies of 350 eV and 580 eV are presented in Figure 2.

The S 2p HRXPS spectra of the target SAMs in Figure 2a are dominated by a characteristic S 2p_3/2,1/2_ doublet at a binding energy (BE) of 162.00–162.05 eV (S 2p_3/2_). This doublet can be clearly assigned to thiolate species bonded to the surface of gold [39–41]. The doublet is the only feature in the spectra of NC-OPE2/Au and NC-OPE3/Au suggesting that all the molecules in these films are bound to the substrate in the SAM fashion, i.e., through the thiolate–gold anchor. In the case of NC-OPE1/Au, this doublet is accompanied by an additional doublet at ~163.5 eV (S 2p_3/2_). This additional feature is associated with a small amount of the physisorbed molecules that are presumably caught in the hydrocarbon matrix or at the SAM-ambient interface, or both. It is quite difficult, or probably even impossible, to get rid of these species in the case of phenylthiolate SAMs on Au [42–43]. The intensity of the thiolate-related doublet in the NC-OPE*n* SAMs decreases with increasing chain length, manifesting a stronger attenuation of the S 2p photoelectrons by the thicker NC-OPE2 and NC-OPE3 films. This is in accordance with the molecular composition and the SAM architecture.

The C 1s HRXPS spectra of NC-OPE1/Au, NC-OPE2/Au and NC-OPE3/Au in Figure 2b are dominated by an intense emission at BEs of 284.4, 284.55, and 284.65 eV, respectively, accompanied by a weaker shoulder at a BE ~1.35 eV higher. The intense emission is related to the OPE backbone, while the high BE shoulder can be assigned to the nitrile carbon. The spectra are mostly representative of the topmost part of the SAMs because of the strong attenuation of the C 1s photoelectrons at the given kinetic energy [41]. In view of this fact, the upward BE shift with the increasing chain length, of both the major emission and the shoulder, is related to a weaker screening of the photoemission hole upon its larger separation from the substrate. This behaviour is distinctly different from the behaviour of the S 2p spectra, in which the position of the thiolate-related doublet is independent of the backbone length. This is understandable, because the location of the thiolate moiety with respect to the substrate does not change with the variation of the backbone length.

The N 1s HXPRS spectra of NC-OPE1/Au, NC-OPE2/Au and NC-OPE3/Au in Figure 2c exhibit a single N 1s emission at BEs of 398.55, 398.85, and 399.10 eV, respectively. This emission is associated with the nitrile groups [30], which are exclusively located at the SAM–ambient interface. The observed BE increase at increasing length of the OPE backbone is similar to that of the C 1s emission and is explained by the same difference in the final state screening. Note that the widths of both of the main emission peaks in the C 1s spectra and in the N 1s spectra decrease with increasing length of the molecular backbone. Most likely, this behaviour reflects a progressive improvement in the orientational and conformational order in the SAMs [41].

Apart from the above qualitative analysis of the HRXPS spectra, we estimated the packing density and effective thickness of the target films on the basis of the HRXPS data. The packing density was estimated by a comparison of the S2p_thiolate_/Au4f intensity ratios of the target films with those for the reference dodecanethiol (DDT) and hexadecanethiol (HDT) systems (a similar approach was successfully used in [44] and [45]). This ratio is a direct measure of the molecular packing density. As compared to the S 2p signal itself, this ratio does not suffer from the problems related to the absolute intensity comparison and to the difference in attenuation of this signal in different films. Due to the quite close binding energies of the Au 4f and S 2p emissions, both signals are attenuated similarly, although not absolutely equally, as far as the primary excitation is performed at high photon energy. The S2p/Au4f intensity ratios for all three target films were found to be quite close to one another (equal within the experimental error) and similar to those for the reference DDT and HDT monolayers. At least for NC-OPE3/Au this agrees with the STM and AFM results, which, as mentioned in the Introduction, suggest that the molecular packing densities in the OPE3 SAMs on Au are close to those of alkanethiol (AT) monolayers [20,35–36].

As for the effective thickness of the target films, this parameter was evaluated on the basis of the C1s/Au4f intensity ratio [46], by assuming a standard expression for the attenuation of the photoemission signal [47], and by using the attenuation lengths reported in [48]. The spectrometer-specific coefficient was calculated on the basis of the analogous procedure performed for the reference DDT and HDT films, the thickness of which is well known [49–50]. By using this approach, the effective thickness of NC-OPE1/Au, NC-OPE2/Au and NC-OPE3/Au was estimated at 13.3, 15.2, and 22.5 Å, respectively. These values can be compared to the corresponding molecular lengths of 7.3, 14.2, and 21.0 Å, which, after the addition of the S–Au spacing (~2.4 Å [51–52]), give the theoretical thickness of the target films for the case of the vertically standing molecules, viz. 9.7, 16.6, and 23.4 Å, respectively. These values suggest a small inclination of the molecules in the target SAMs, which, in view of a limited accuracy of the thickness evaluation procedure, can only be considered as a tentative statement, whereas the exact molecular orientation can be estimated by the NEXAFS spectroscopy (see the following section). Note, however, that whereas the theoretical thicknesses of the NC-OPE2 and NC-OPE3 films are lower than the values derived from the experiment, the opposite is true for the NC-OPE1 SAMs. This suggests, in accordance with the S 2p spectrum for these SAMs (Figure 2), the presence of a certain amount of the physisorbed molecules at the SAM-ambient interface in the case of NC-OPE1/Au.

### NEXAFS spectroscopy

NEXAFS spectroscopy samples the electronic structure of unoccupied molecular orbitals and provides information about the integrity and chemical identity of the adsorbed film. In many cases, NEXAFS spectroscopy allows a better distinction between different chemical species and functional groups as compared to HRXPS and, in this regard, is a complementary technique. The chemical information is best represented by a spectrum acquired at the so-called magic angle of X-ray incidence (55°); this spectrum is not affected by any effects related to molecular orientation and is only representative of the chemical identity of investigated samples [53]. Furthermore, by using the angular dependence of the transition-matrix elements for resonant excitations [53], the average orientation of the film constituents can be derived from the NEXAFS experiment. A fingerprint of such an orientation is the linear dichroism (see Experimental section), which, among other means, can be efficiently monitored by plotting the difference between the NEXAFS spectra acquired at normal (90°) and grazing (20°) angles of X-ray incidence.

**Figure 3:**
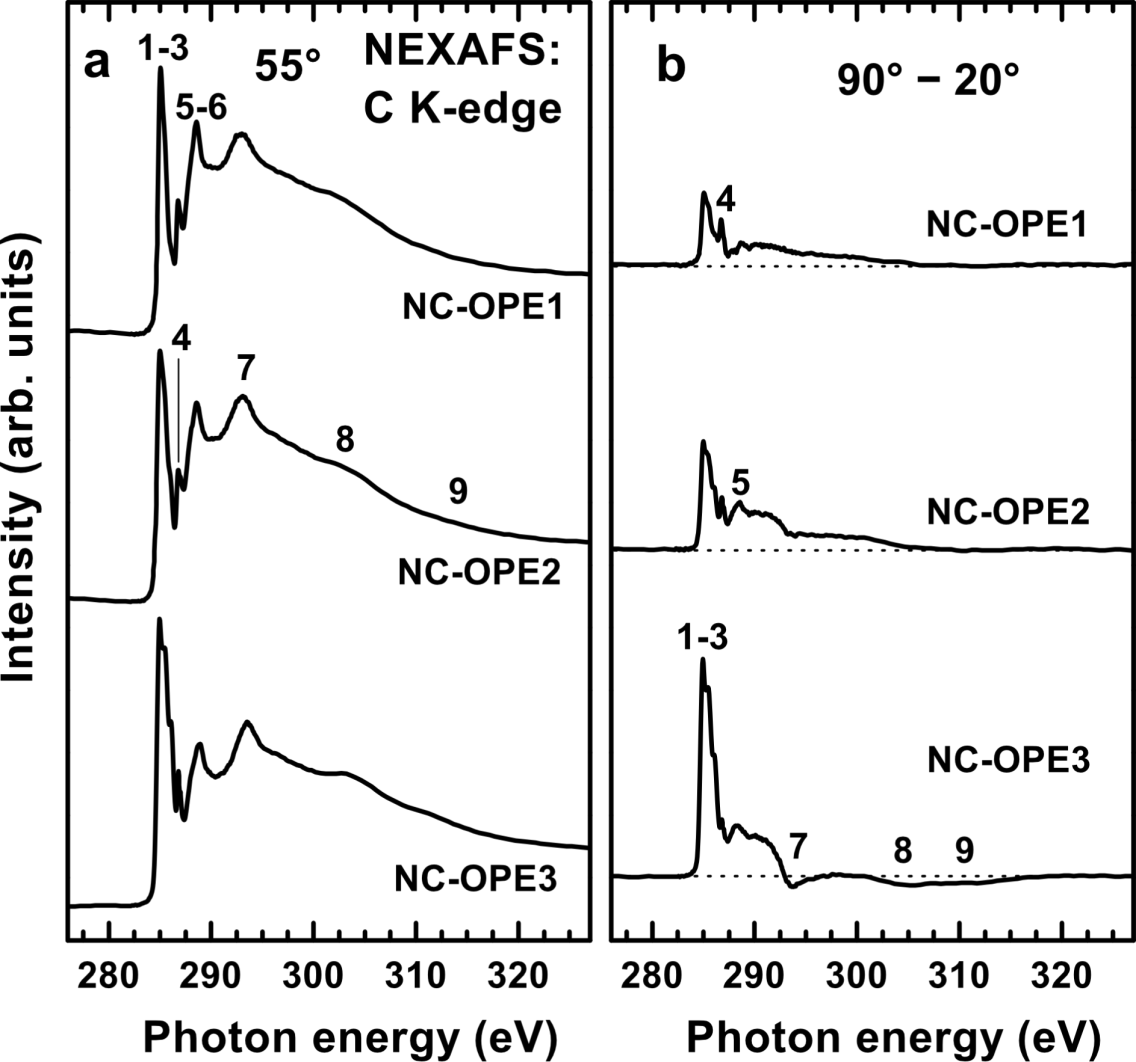
(a) C K-edge NEXAFS spectra of the NC-OPE*n* SAMs acquired at an X-ray incidence angle of 55°. (b) Difference between the C K-edge spectra acquired at X-ray incidence angles of 90° and 20°. The zero level of the difference spectra is shown by dotted lines. The most prominent absorption resonances are marked by numbers; see text for details.

**Figure 4:**
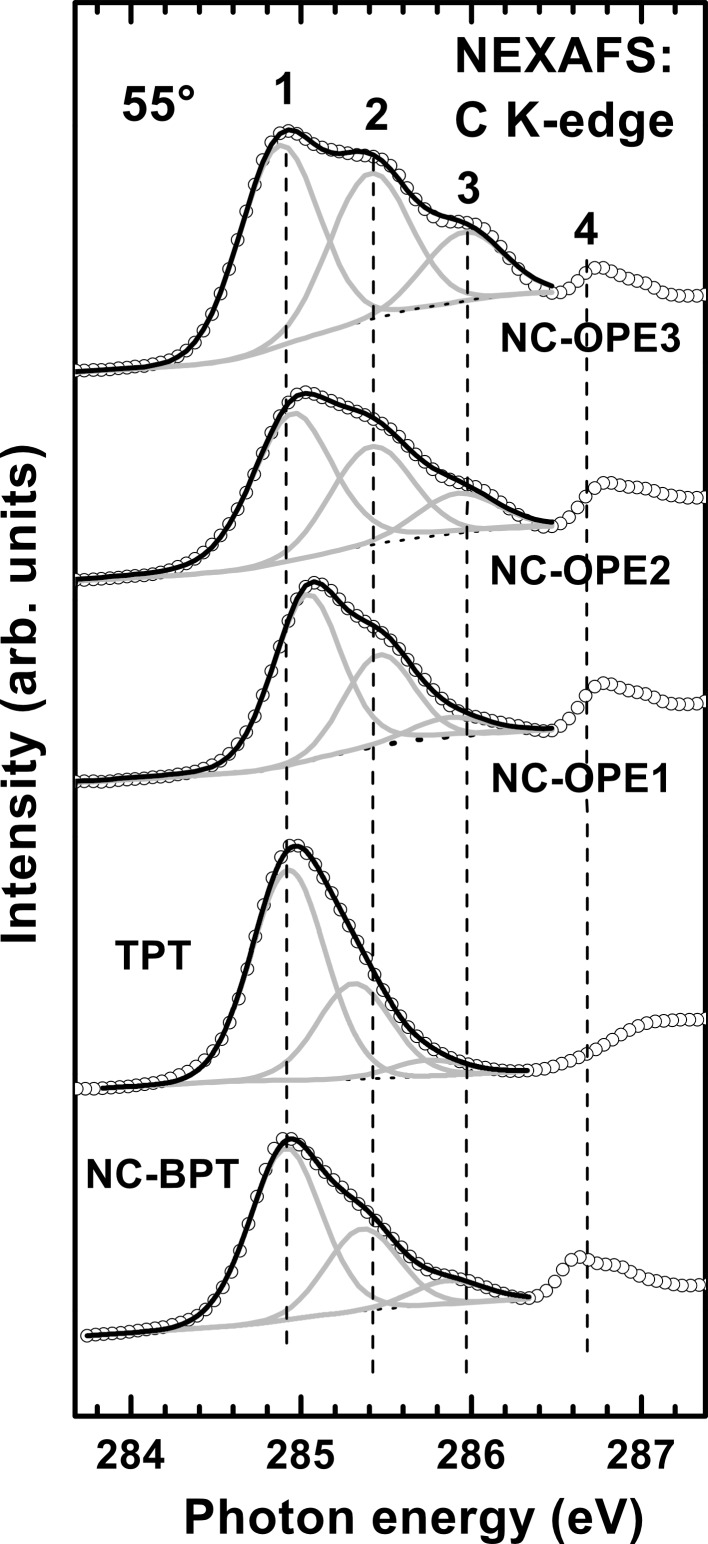
π*-resonance photon-energy range of the C K-edge NEXAFS spectra of the target SAMs and two reference films, NC-BPT/Au and TPT/Au. The spectra are decomposed into the individual contributions, which are marked by numbers; see text for details.

The C K-edge NEXAFS spectra of the NC-OPE*n* SAMs acquired at an X-ray incidence angle of 55° are presented in Figure 3a, whereas the π*-resonance photon-energy range of these spectra is shown in detail in Figure 4, along with the spectra of the two reference systems, viz. SAMs of nitrile-substituted biphenylthiol (NC-BPT) [30] and 1,1′;4′,1″-terphenyl-4-thiol (TPT) [42–43] on Au. The spectra of the target films are dominated by a strong peak, consisting of at least three absorption resonances at 284.9–285.0 eV (**1**), 285.40–285.45 eV (**2**), and 286.0 eV (**3**); see Figure 4. The resonances **1** and **3** can be assigned with certainty to the π_1_* orbital of the aromatic rings and to the π*(C≡C) orbital [53], respectively, and this is additionally supported by the intensity increase of the latter resonance with the increasing chain length. The resonance **2** is presumably comprised of several different contributions, including a conjugation between the π* orbital of the rings and C≡C groups [53]. There are also contributions from the phenyl rings themselves, as seen in the spectrum of TPT/Au in which a tentative decomposition of the asymmetric resonance is performed (note that the asymmetry is related to the vibrational structure of the resonance) [53].

In addition to the joint resonance **1**–**3**, a comparatively sharp resonance at 286.75 eV (**4**) is observed in the spectra of all the NC-OPE*n* films. This resonance can be, with certainty, associated with the nitrile group since it has exactly the same energy as the characteristic π* resonance of nitrile in the films of nitrile-substituted alkanethiolates [31–32,54] and oligophenyls [30,33]. In particular, this resonance is clearly seen in the spectrum of NC-BPT, as shown in Figure 4. At the same time, this resonance is not observed in the spectra of nonsubstituted OPEs [37] and oligophenyls [42,55], including the spectrum of TPT/Au shown in Figure 4.

Along with the above-mentioned features, there are several further resonances at 288.1 eV (**5**), 288.7 eV (**6**), 293.6 eV (**7**), ~304.6 eV (**8**), and ~311.0 eV (**9**); these resonances are marked by numbers in Figure 3. The respective molecular orbitals have either π* character (**5** and **6**) or σ* character (**7**–**9**) [42,53–54,56].

**Figure 5:**
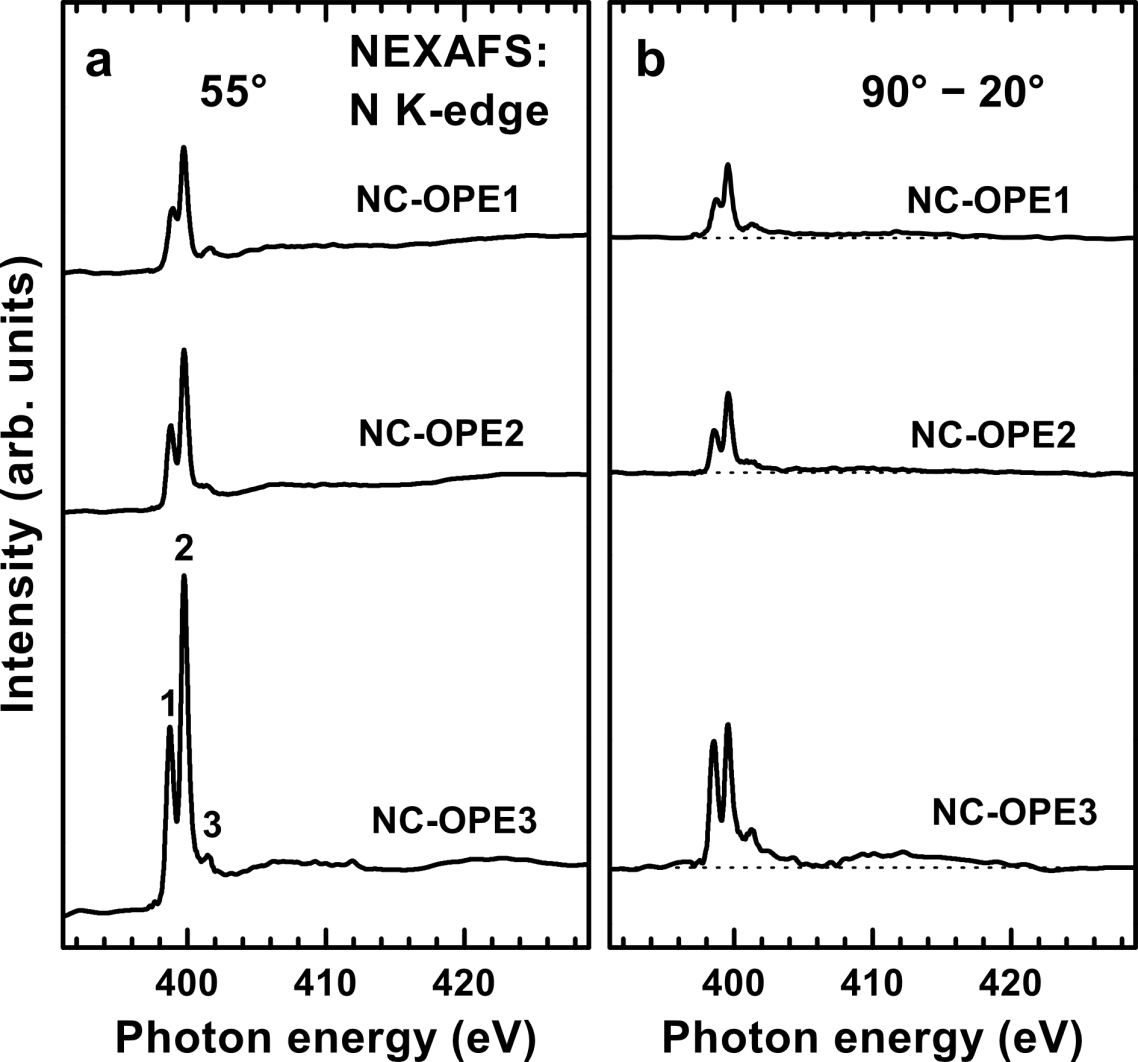
(a) N K-edge NEXAFS spectra of the NC-OPE*n* SAMs acquired at an X-ray incidence angle of 55°. (b) Difference between the N K-edge spectra acquired at X-ray incidence angles of 90° and 20°. The zero level of the difference spectra is shown by dotted lines. The most prominent absorption resonances are marked by numbers; see text for details.

The N K-edge NEXAFS spectra of the NC-OPE*n* SAMs acquired at an X-ray incidence angle of 55° are presented in Figure 5a. A dominant feature in these spectra is a characteristic double resonance at ~398.80 eV (**1**) and ~399.75 eV (**2**); it is accompanied by several weaker features, including a π*-character resonance at ~401.5 eV and several σ*-character resonances at higher photon energy. These spectra resemble that of benzonitrile [57–58] and are also typical of SAMs containing this moiety [29–30,33,59]. The appearance of the dominant double resonance is caused by the conjugation between the π* orbitals of the nitrile group and those of the adjacent phenyl ring. Due to such a conjugation, the degeneracy of the π* orbitals of the nitrile group is lifted, and they split into two states with different energies. One of the resulting orbitals (lower photon energy; π_1_* or **1**) is oriented perpendicular to the ring plane; the another one (higher photon energy; π_3_* or **2**) is parallel to this plane [33,57–58]. Due to the delocalization of the π_1_* orbital over the entire benzonitrile moiety, the intensity of the π_1_* resonance is lower as compared to the π_3_* resonance (the orbital is almost exclusively localized on the nitrile group) [30,33]. Note that the π* resonance of the nitrile group splits not only at the N but also at the C K-edge (see Figures S3 and S4 in Supporting Information File 1). However, since there is only one carbon atom in the nitrile group, the respective split resonance has a relatively low intensity in the C K-edge spectra. As a result, only π_3_*(CN) is clearly visible (resonance **4** in Figure 4; see [30]), whereas the even weaker π_1_*(CN) resonance overlaps with the resonance **3** (Figure 4) and is practically imperceptible.

**Figure 6:**
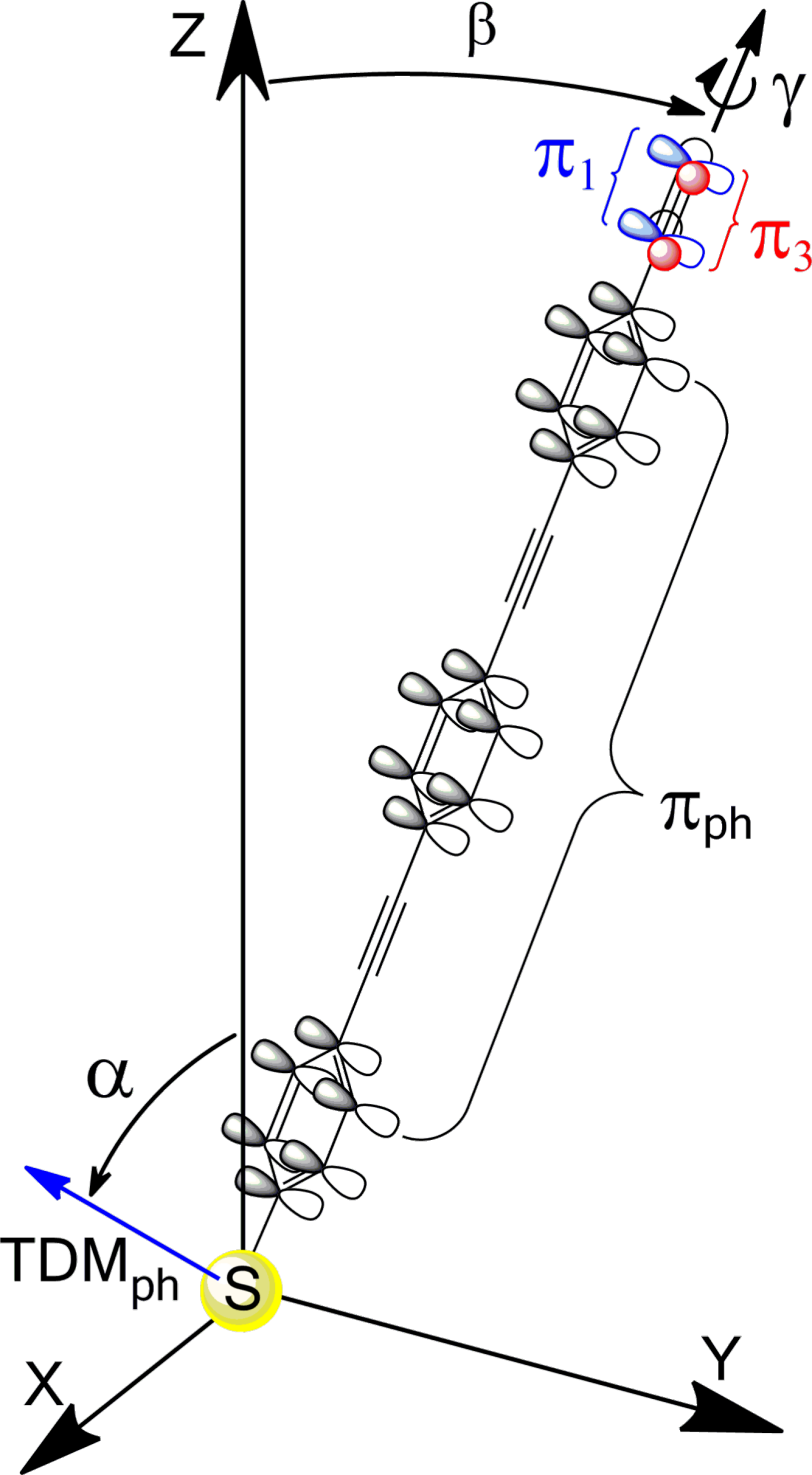
Orientation of the NC-OPE*n* molecules in the respective SAMs (by the example of NC-OPE3; a planar conformation is assumed). The orientation of the molecular backbone is given by the tilt angle β (tilt within the z–y plane) and twist angle γ. The π* orbitals of the phenyl rings (π_ph_*), constituting the backbone, are perpendicular to the ring plane, with the orientation of TDM_ph_ (blue arrow) given by the tilt angle α. π_1_* (blue) and π_3_* (red) orbitals of the nitrile group are perpendicular and parallel to the plane of the adjacent phenyl ring, respectively.

Along with the above results, the NEXAFS data provide information on the orientation of the molecular constituents in the target films. Both C and N K-edge spectra of the target SAMs exhibit significant linear dichroism as follows from the differences between the spectra acquired at normal and gracing (20°) incidence of the primary X-ray beam shown in Figure 3b and Figure 5b. The difference peaks related to the π* resonances are distinctly positive, which, in view of the orientation of the transition dipole moments (TDMs) associated with these resonances (perpendicular to the molecular backbone), suggests an upright orientation of the target molecules in the SAMs. A schematic drawing of this orientation is shown in Figure 6, through the example of NC-OPE3, which presumably takes a planar conformation in the densely packed SAM (see below). The π* orbitals of the phenyl rings (π_ph_*) are perpendicular to the molecular plane; the respective TDM_ph_, which is perpendicular to the molecular plane as well, is shown as a blue arrow. π_1_* (blue) and π_3_* (red) orbitals of the nitrile group are perpendicular and parallel to the molecular plane, respectively. The molecular orientation is described by the tilt (β) and twist (γ) angles of the molecular backbone. The molecular tilt occurs within the z–y plane. The twist is defined in terms of γ = 0 when TDM_ph_ lies in the plane spanned by the z- and the molecular axes (i.e., in the z–y plane).

For the nonsubstituted aromatic and OPE SAMs, β and γ cannot be strictly evaluated on the basis of the NEXAFS data. These data only provide information on the average orientation of the TDM_ph_, given by the tilt angle α (Figure 6), whereas the value of β can only be calculated as far as a reasonable assumption about the molecular twist can be made [60–61], e.g., on the basis of the molecular orientation in the respective bulk materials. This situation changes, however, in the case of the nitrile substitution due to the presence of the π_1_* and π_3_* orbitals of the nitrile group, which are perpendicular to each other and one of which is aligned with the π_1_* orbitals of the phenyl rings. In this case, both β and γ can be directly derived from the NEXAFS data at the N K-edge from a system of nonlinear equations

[1]
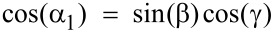


[2]
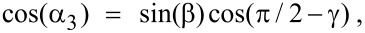


where α_1_ and α_3_ are the average tilt angles of the π_1_* and π_3_* orbitals of the nitrile group, respectively [29]. These angles can be derived from the evaluation of the entire set of the N K-edge NEXAFS spectra taken at different angles of X-ray incidence, θ, according to the standard equation for the intensity of a vector-type orbital [53]

[3]
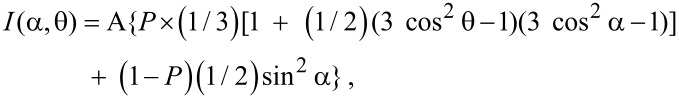


where I(α,θ) is the intensity of either the π_1_* or π_3_* resonance, *A* is a constant, and *P* is the polarization factor of the synchrotron light. The resulting values of α_1_ and α_3_ are given in Table 1. By using these values, the average twist angle of the OPE backbone in the NC-OPE SAMs can be directly calculated from equation

[4]
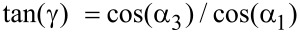


obtained from the division of Equation 2 by Equation 1. Equal values of α_1_ and α_3_, as are found for NC-OPE1/Au and NC-OPE2/Au, mean thus that γ is close to 45°. A higher value of α_1_ as compared to α_3_, as is the case for NC-OPE3/Au, means that γ is larger than 45°. The derived values of γ presented in Table 1 are in accordance with these qualitative considerations. Furthermore, using either Equation 1 or Equation 2, the average tilt angle of the OPE backbone in the NC-OPE*n* SAMs can be calculated, and the respective values are given in Table 1; they are close to each other for all target SAMs, independent of the chain length. Note that this result is somewhat in contrast to the C K-edge spectra in Figure 3, which exhibit an increasing linear dichroism with increasing length of the molecular chain in NC-OPE*n*/Au. This dichroism can be presumably associated with the improved orientational order on going from NC-OPE1/Au to NC-OPE2/Au and further to NC-OPE3/Au.

**Table 1:** Derived average tilt angles for the π_1_*and π_3_* orbitals of the nitrile group (from Equation 3) as well as twist and tilt angles for the OPE backbone in the NC-OPE*n* SAMs on Au(111). The absolute accuracy of the angle values is ±3°, which are the standard error bars in the case of NEXAFS spectroscopy. The relative accuracy is noticeably higher.

Film	NC-OPE1	NC-OPE2	NC-OPE3

**α_1_**	67.4°	65.5°	70.2.°
**α_3_**	67.4°	65.1°	62.9°
**γ**	44.9°	45.3°	53.3°
**β**	33.0°	36.3°	34.5°

Note that we assumed a planar conformation of the OPE backbone for NC-OPE2/Au and NC-OPE3/Au within the analysis of the molecular orientation. We expect this conformation for the densely packed NC-OPE*n* monolayers (see next section), similar to the SAMs with oligophenyl backbone, for which the individual rings are twisted differently (torsion) in the molecular state but adapt to a planar conformation in the monolayer state [62]. According to our estimates, the barrier for adapting to a planar conformation is much lower in the case of OPE as compared to that of oligophenyl, as far as no side functionalization of the individual rings along the OPE backbone is performed.

### Calculation of the NEXAFS spectra

We calculated the NEXAFS spectra of the OPE3 (Supporting Information File 1) and NC-OPE3 molecules in two different conformations, viz. in the planar conformation, with all three phenyl rings located in the same plane, and in a twisted conformation, with the central ring rotated by 90° with respect to the two other rings about the molecule axis. Note that the latter conformation may occur in the gaseous phase while the former is expected to be preferred for the densely packed molecular assembles, such as bulk samples and SAMs.

**Figure 7:**
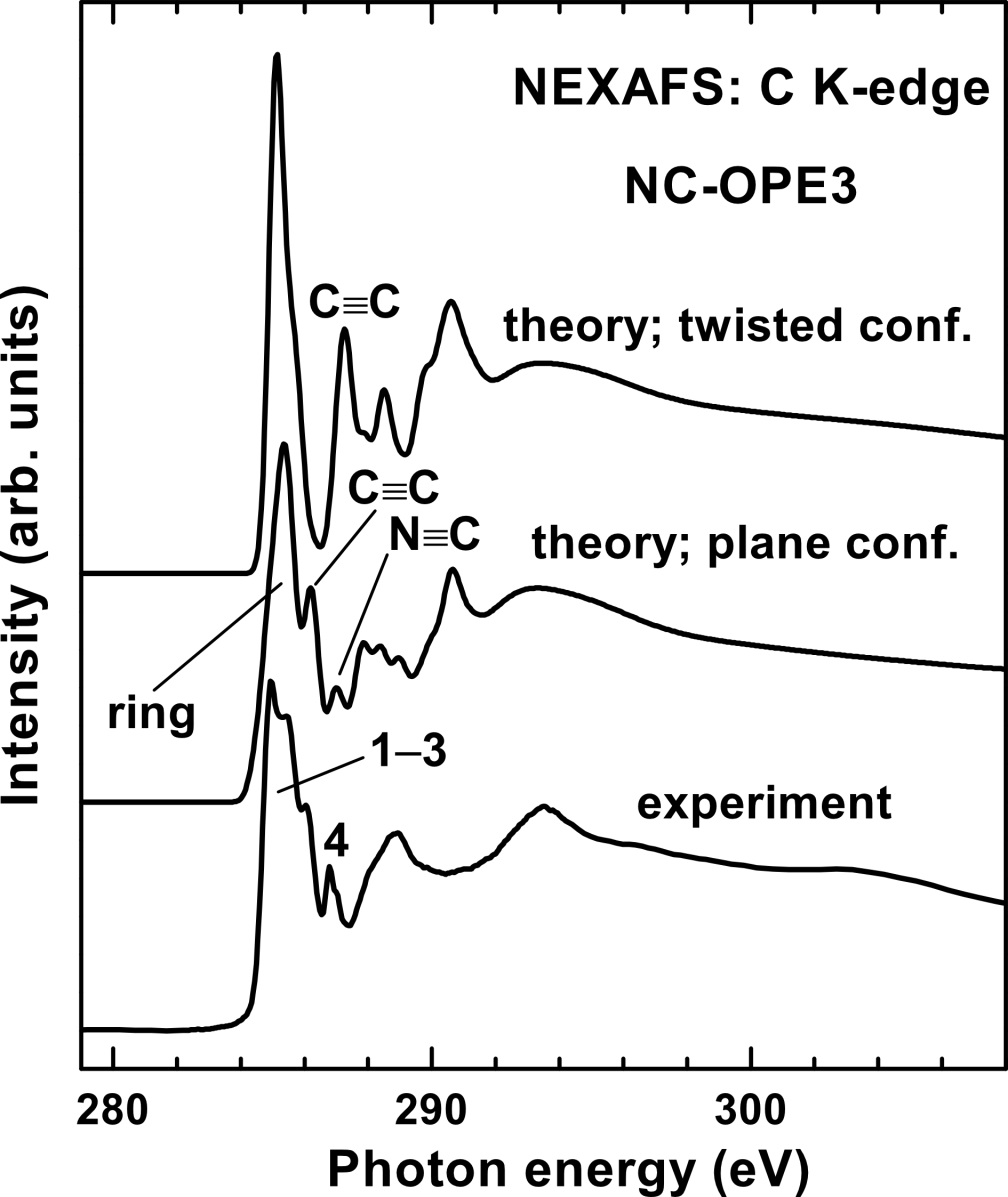
Calculated C K-edge NEXAFS spectra of NC-OPE3 in the planar and twisted conformations, along with the experimental spectrum of NC-OPE3/Au taken at an X-ray incidence angle of 55°. The theoretical spectra were shifted by ca. 1.3 eV to lower photon energies in order to align the most intense π* resonances in the theoretical and experimental spectra. The most prominent absorption resonances in the experimental spectrum are marked by numbers. The most prominent absorption resonances in the theoretical spectrum are marked by the functional groups that are associated with these resonances.

**Figure 8:**
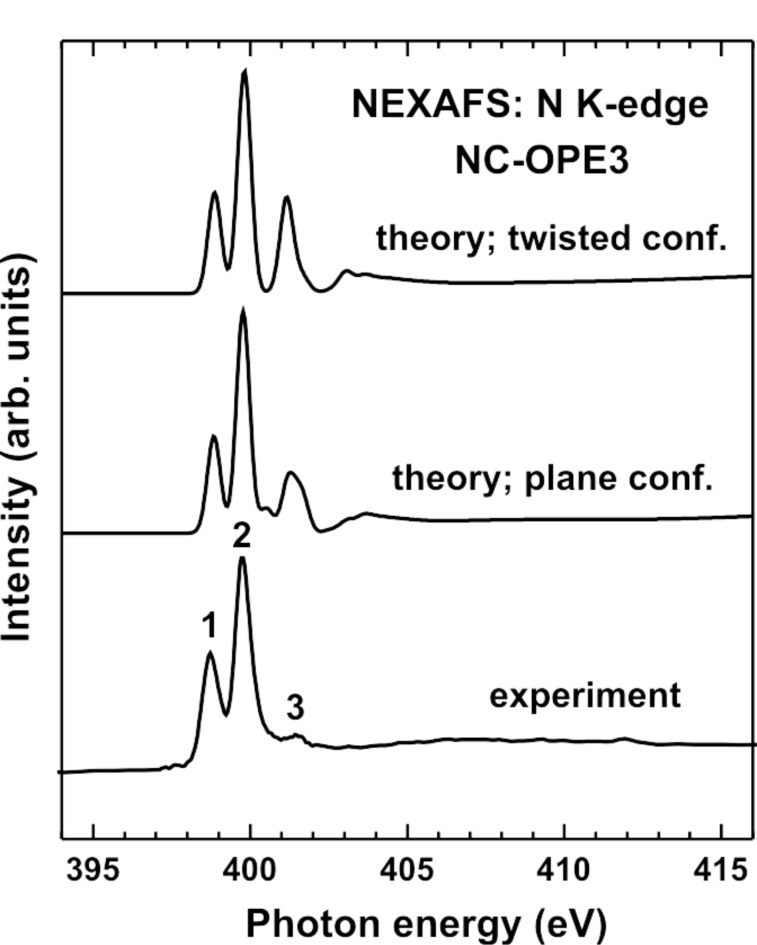
Calculated N K-edge NEXAFS spectra of NC-OPE3 in the planar and twisted conformations, along with the experimental spectrum of NC-OPE3/Au taken at an X-ray incidence angle of 55°. The theoretical spectra were shifted by ca. 2.3 eV to lower photon energies in order to align the most intense π* resonances in the theoretical and experimental spectra. The most prominent absorption resonances in the experimental spectrum are marked by numbers.

Calculated C and N K-edge NEXAFS spectra of NC-OPE3 in the planar and twisted conformations are presented in Figure 7 and Figure 8, respectively, along with the corresponding experimental spectrum of NC-OPE3/Au taken at an X-ray incidence angle of 55°. The theoretical C K-edge spectra represent sums over the separately calculated partial spectra of the 17 different carbon atoms in the NC-OPE3 molecule, which allows identification of the contribution of each of the different functional groups to the individual resonances. The three major functional groups are the phenyl rings, the C≡C group, and the nitrile moiety. Whereas the exact decomposition of the theoretical spectra can be found in Supporting Information File 1, we assigned the most prominent absorption resonances in Figure 7 in accordance with the functional groups that provide the major contribution to these resonances. Taking into account these assignments and comparing the theoretical and experimental data, we can conclude that the theoretical spectrum for the planar conformation of NC-OPE3 reproduces the experimental spectrum of NC-OPE3/Au much better than does the calculated curve for the twisted conformation of NC-OPE3. In addition, this comparison supports our assignment of the most prominent absorption resonances: **1** as related to the phenyl rings; **3** to the C≡C groups; **2** to the conjugation of the above two moieties; and **4** to the nitrile group. Interestingly, the molecular orbitals associated with the resonance **2** are mostly located on the phenyl rings.

The theoretical N K-edge spectra of NC-OPE3 in Figure 8 reproduce perfectly the experimental result, both from the viewpoint of the resonant pattern and of the relative intensity of the most prominent π_1_* and π_3_* features. However, similar to the C K-edge data, the theoretical spectrum for the planar conformation of NC-OPE3, which exhibits much lower relative intensity of the resonance **3**, reproduces the experimental spectrum of NC-OPE3/Au much better than does the calculated curve for the twisted conformation of NC-OPE3. This supports our above conclusion about the planar molecular conformation of NC-OPE3/Au in the respective SAMs. Note that the same conformation can also be expected for NC-OPE2/Au.

## Discussion

Both HRXPS and NEXAFS data suggest consistently that the NC-OPE*n* SAMs on Au(111) are well-defined and contamination-free, apart from a minor portion of physisorbed molecules in NC-OPE1/Au, with the SAM molecules bound to the substrate through the gold–thiolate anchor and the nitrile tail groups exclusively located at the SAM–ambient interface. The HRXPS data show that independent of the chain length, all of the SAMs have similar packing densities, which, in accordance with the literature data [20,35–36], are quite close to those of AT SAMs on Au(111). Such packing density likely means that a herring-bone type of motif exists, which is the typical configuration for both bulk aromatic materials (see, e.g., [63]) and their respective monolayers [62,64–65].

Similar to the SAMs with a nonsubstituted OPE backbone [42–43], orientational order in NC-OPE*n* films depends on the length of the molecular chain, improving with increasing chain length according to the C K-edge NEXAFS data. At the same time, molecular inclination of the SAM constituents in the NC-OPE*n* SAMs is almost independent of the chain length, with an average tilt angle of ~33–36°. Interestingly, the twist angle of the OPE backbone, which exhibits a fully planar conformation for the SAMs (all three rings in the same plane), is identical for the NC-OPE1 and NC-OPE2 SAMs at 45°, similar to the case of the nonsubstituted oligophenyl backbone (34.5–41.2° [29]), whereas it is higher for the NC-OPE3 SAM at 53.5°. According to a previous detailed IRS analysis, OPE3 SAMs with no terminal group exhibit an average molecular tilt of 33 ± 18° from the surface normal [36], which correlates well with our value of 34.5° for NC-OPE3/Au. Further, the average twist angle in the OPE3 films was found to be 31 ± 6° [36], which, when converted to match our definition of twist angle, is equivalent to 59° and hence is very close to our value of 53.3° for NC-OPE3/Au [66]. From this comparison it is clear that substitution of the OPE3 backbone by the nitrile group does not affect the molecular orientation significantly. This is in contrast to the aliphatic NC-terminated SAMs in which the introduction of the nitrile tail group results in a significant disturbance of the molecular orientation and orientational order [54]. This disturbance can be understood in terms of the strong electrostatic interactions between the nitrile groups, bearing a large dipole moment of 3.9 D [67], which will provide electrostatic stresses when neighbouring dipoles have unfavourable alignments. In the case of the flexible aliphatic backbone, the stresses can be relieved in part by inducing strains, primarily through the appearance of *gauche* defects at the terminal –CH_2_– units of the alkyl chain. Such conformational changes, however, are not possible in the case of rigid oligophenyl or OPE backbone, which leads to a certain persistence of the molecular lattice even in the case of the strongly interacting tail groups.

It is interesting to compare the NC-OPE*n* monolayers with the respective systems without the triple bonds. The closest systems are the NC-BPT SAMs [29–30] and the monolayers of 4″-(mercaptomethyl)terphenyl-4-yl-carbonitrile, NC–(C_6_H_4_)_3_–(CH_2_)–SH (NC-TP1) [29] (regrettably, there are no published data for the closest system, NC–(C_6_H_4_)_3_–SH, abbreviated as NC-TPT). The NC-BPT SAMs on Au(111) are characterized by an average tilt angle of ~39° and a twist angle of 40.8° [29]. These values are quite close to the analogous values for NC-OPE2 SAMs (36.3° and 45.3°, respectively). The molecular tilt in the latter system is slightly smaller, presumably due to a longer molecular backbone, whereas the twist is higher. Analogously, the NC-TP1 SAMs on Au(111) are characterized by an average tilt angle of ~34.0° and a twist angle of 47.1°. Once more, these values are quite close to the analogous values for the NC-OPE3 SAMs (34.5° and 53.3°, respectively). Considering that the introduction of the methylene linker results in a lesser molecular inclination in the terphenyl-based SAMs [68], we could assume that the molecular tilt in the NC-OPE3 SAMs is smaller than that in the NC-TPT monolayers; this is once more a clear effect of the molecular backbone length. The twist angle for the NC-OPE3 SAMs is higher than that for the NC-TP1 monolayers and presumably even higher than that for the NC-TP0 film (on the basis of the values for the biphenyl-based SAMs [29]). In summary, the introduction of the –C≡C– groups into the oligophenyl backbone results in an expected slight decrease of molecular inclination (chain-length effect) and a noticeable increase of molecular twist. The latter can be of importance for understanding of the exact molecular arrangement in the OPE SAMs.

## Conclusion

We presented here the results of the spectroscopic characterization for a series of nitrile-substituted thiolated OPEs assembled in the SAM fashion on Au(111). This characterization included the synchrotron-based complementary techniques of HRXPS and angle-resolved NEXAFS spectroscopy at both C and N K-edges, which were additionally supported by quantum-mechanical calculations of the NEXAFS spectra. The length of the OPE chain in the SAMs was varied from one to three structural units to test the effect of the chain length on the integrity, packing density, and molecular orientation of the SAMs. The nitrile tail group serves as a distinct spectroscopic marker for X-ray absorption measurements, which allowed us to probe directly both the molecular tilt and twist.

The experimental data suggest that the NC-OPE*n* molecules form well-defined and contamination-free SAMs on Au(111). Apart from a minor proportion of physisorbed molecules in NC-OPE1/Au, all molecules in these SAMs are bound to the substrate over the gold-thiolate anchor, whereas the nitrile tail groups are exclusively located at the SAM–ambient interface. Independent of the chain length, all the SAMs have similar packing densities, which are quite close to those of AT SAMs on Au(111). Whereas the orientational order in NC-OPE*n* films depends on the length of the molecular chain, improving with increasing chain length, the molecular inclination of the SAM constituents is almost independent of the chain length, with an average tilt angle of ~33–36°. At the same time, the twist of the OPE*n* backbone was found to depend on the molecular length, being close to 45° for NC-OPE1/Au and NC-OPE2/Au, but ~53.5° for NC-OPE3/Au. Comparison of the molecular orientation in the NC-OPE3/Au system with the literature data for the analogous nonsubstituted film suggests that the attachment of nitrile to the OPE3 backbone does not significantly affect the molecular orientation in the SAMs. This was explained by the rigidity of the OPE3 backbone and stability of the densely packed molecular lattice, which consists of OPE3 moieties in planar conformation arranged, presumably, in a herring-bone fashion.

The results of this study provide important data that are relevant to the use of these types of “molecular wires” for applications in molecular-electronics devices, particularly with regard to studies of the dynamics of charge-transport behaviour.

## Experimental

The NC-OPE*n* compounds were synthesized according to previous protocols [69]. The purity of all the compounds was checked by NMR. The gold substrates were prepared by thermal evaporation of 100–200 nm of gold (99.99% purity) onto polished single crystal silicon (100) wafers (Silicon Sense) primed with either a 5 nm titanium or a 5 nm chromium adhesion layer. The evaporated films were polycrystalline, with a predominant (111) texture [40,70] and grain sizes of 20–50 nm. To prepare the SAMs, these substrates were immersed into a 1 mmol solution of the NC-OPE*n* compounds in toluene or in methylene chloride for 24 h at room temperature, with identical results in either solvent. Afterwards, the SAM samples were carefully rinsed by immersion in the solvent and further rinsing with absolute ethanol. Finally, they were blown dry with argon or nitrogen gas.

In addition to the OPE SAMs of interest, several reference SAMs were prepared on Au(111) substrates using standard procedures. The reference SAMs included those formed from DDT [50], HDT [71], TPT [42–43], and NC-BPT [30].

The SAMs were characterized by several complementary spectroscopic techniques, viz., high-resolution X-ray photoelectron spectroscopy (HRXPS), angle-resolved near-edge X-ray absorption fine-structure (NEXAFS) spectroscopy and infrared reflection spectroscopy (IRS). The HRXPS and NEXAFS spectroscopy experiments were conducted at the bending magnet beamline D1011 (plane-grating monochromator) of the synchrotron storage ring MAX II at MAX-Lab in Lund, Sweden. We used an experimental station equipped with a SCIENTA SES200 electron-energy analyzer and a partial-electron-yield (PEY) detector. The experiments were carried out under UHV conditions at a base pressure <1.5 × 10^−10^ mbar. We took care to avoid any noticeable damage induced by X-rays [72–75], minimizing the spectra acquisition time and performing control measurements on reference samples.

The HRXPS spectra were collected in normal emission geometry. Photon energy (PE) was varied; it was set at 350 eV for the S 2p region, at 350 and 580 eV for the C 1s range, and at 580 eV for the N 1s and O 1s regions. The BE scale of every spectrum was individually calibrated with reference to the Au 4f_7/2_ emission line of the substrate at 83.95 eV [76]. For this purpose, Au 4f spectra were acquired for each sample and at each PE change. The energy resolution was better than 100 meV, which is noticeably smaller than the full widths at half maximum (fwhm) of the photoemission peaks of the S 2p, C 1s, and N 1s spectra.

HRXPS spectra were fitted by symmetric Voigt functions and either a Shirley-type or linear background. To fit the S 2p_3/2,1/2_ doublets we used a pair of such peaks with the same fwhm, a branching ratio of 2 (2p_3/2_/2p_1/2_), and spin-orbit splittings (verified by fit) of ~1.18 eV (2p_3/2_/2p_1/2_) [77]. The fits were carried out self-consistently: The same peak parameters were used for identical spectral regions. The accuracy of the resulting BE/fwhm values is 0.02–0.03 eV.

The NEXAFS spectra were acquired at the carbon and nitrogen K-edges. We used the partial-electron-yield acquisition mode with retarding voltages of −150 and −300 V for the C and N K-edges, respectively. Primary X-ray beam was linearly polarized with a polarization factor of ~95%. The energy resolution was less than 100 meV. To monitor the orientational order of the target molecules within the films, the incidence angle of the X-ray beam was varied from 90° (**E**-vector in the surface plane) to 20° (**E**-vector nearly normal to the surface) in steps of 10–20°. This approach is based on the strong dependence of the cross-section of the resonant photoexcitation process on the orientation of the electric field vector of the linearly polarized light with respect to the molecular orbital of interest [53]. This effect is usually described as linear dichroism in X-ray absorption [53]. The accuracy of the incidence-angle adjustment was ±0.5°.

The raw spectra were normalized to the incident photon flux by division by a spectrum of a freshly sputtered, clean gold sample and were reduced to the standard form [53]. The energy scale was calibrated to the most intense π* resonance of highly oriented pyrolytic graphite at 285.38 eV [78] in combination with the well-known Δ(hν) 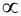 (hν)^3/2^ behaviour of plane grating monochromators [79]. The resultant energy positions are expected to be accurate and reproducible within ±0.05 eV.

In order to provide a reliable basis for the assignment of the features in the experimental NEXAFS spectra and to get information about the molecular conformation in the target SAMs, a series of calculations with the quantum-chemistry program package StoBe (**Sto**ckholm-**Be**rlin) [80] were carried out for the OPE3 and NC-OPE3 molecules. Note that StoBe is used to evaluate and analyze the electronic structure as well as spectroscopic and other properties of molecules and atom clusters. The approach is based on self-consistent solutions of the Kohn-Sham equations employing linear combinations of Gaussian type orbitals. The theory and numerical details of the realization can be found in [80–83]. As a further verification of the integrity of the SAMs, infrared spectra were obtained. In all cases the SAMs had the expected spectra based on reference spectra of the pure thiol molecules used for self-assembly.

## Supporting Information

Supporting Information features the calculated C and N K-edge spectra of OPE3 and NC-OPE3 in the planar and twisted conformation, decomposed into the partial spectra related to the individual building blocks of the target molecules.

File 1 Calculated X-ray absorption spectra.
