# Supplementary material for: X-ray spectroscopy characterization of self-assembled monolayers of nitrile-substituted oligo(phenylene ethynylene)s with variable chain length
Source: Beilstein J Nanotechnol. 2012 Jan 5;3:12–24. doi: 10.3762/bjnano.3.2 (PMC3304316; doi:10.3762/bjnano.3.2)
Supplement: File 1 — Calculated X-ray absorption spectra. [file Beilstein_J_Nanotechnol-03-12-s001.pdf]

Supporting Information

for

X-Ray spectroscopy characterization of self-assembled monolayers of nitrile-substituted oligo(phenylene ethynylene)s with variable chain length

Hicham Hamoudi,[1] Ping Kao,[2] Alexei Nefedov,[3] David L. Allara,[2] and Michael Zharnikov*,[1,§]

Address: [1]Angewandte Physikalische Chemie, Universität Heidelberg, D-69120 Heidelberg, Germany; [2]Departments of Chemistry and Material Science, Pennsylvania State University, University Park, PA16802, USA and [3]Institut für Funktionelle Grenzflächen, Karlsruher Institut für Technologie, Eggenstein-Leopoldshafen, D-76344, Germany

Email: Michael Zharnikov* - Michael.Zharnikov@urz.uni-heidelberg.de

*Corresponding author

[§]Phone: +49-6221-54 4921, Fax: +49-6221-54 6199

Calculated X-ray absorption spectra

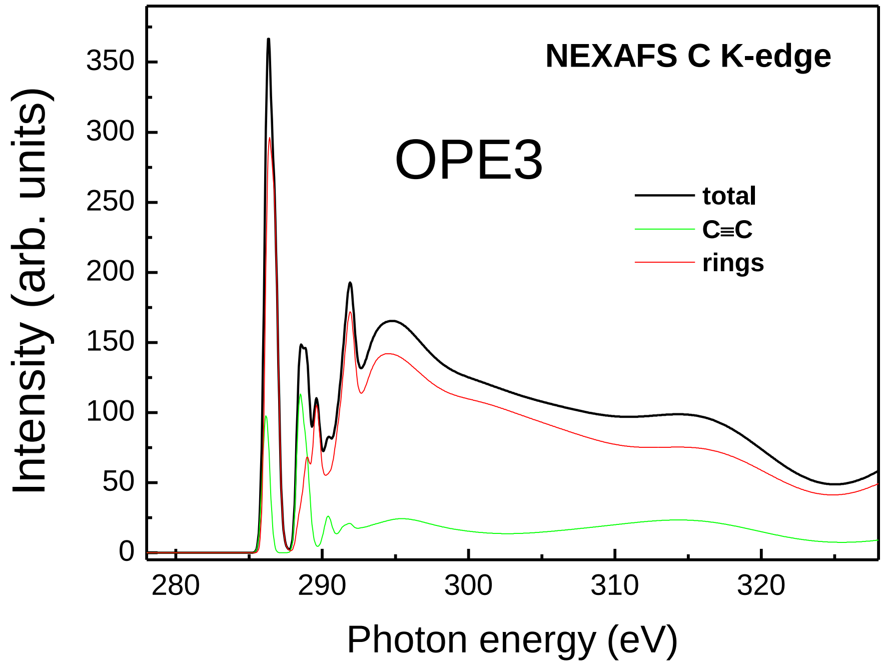

Figure S1: Calculated C K-edge NEXAFS spectra of OPE3 in the twisted conformations. The spectrum is decomposed into the contributions related to the individual functional units.

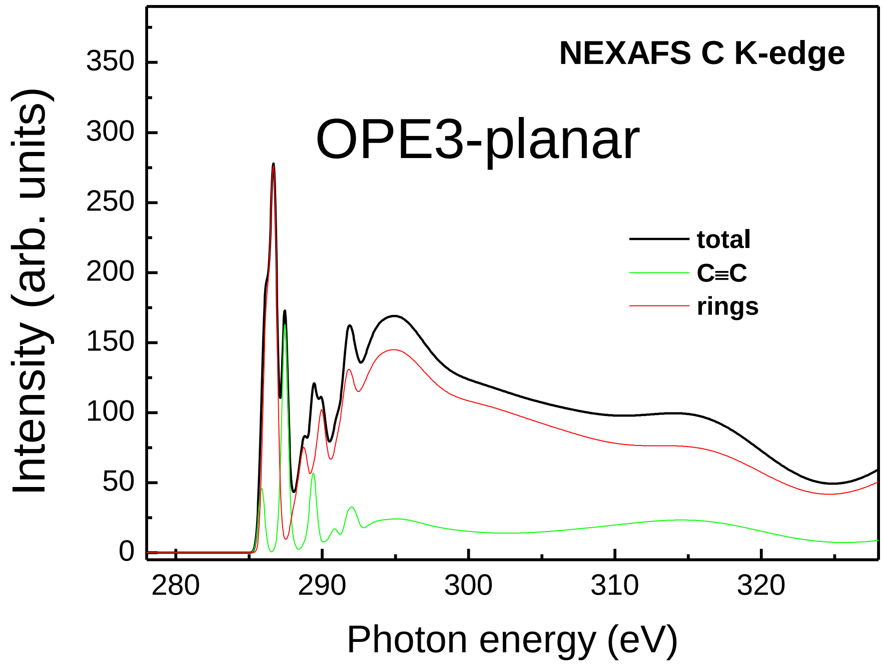

Figure S2: Calculated C K-edge NEXAFS spectra of OPE3 in the planar conformations. The spectrum is decomposed into the contributions related to the individual functional units.

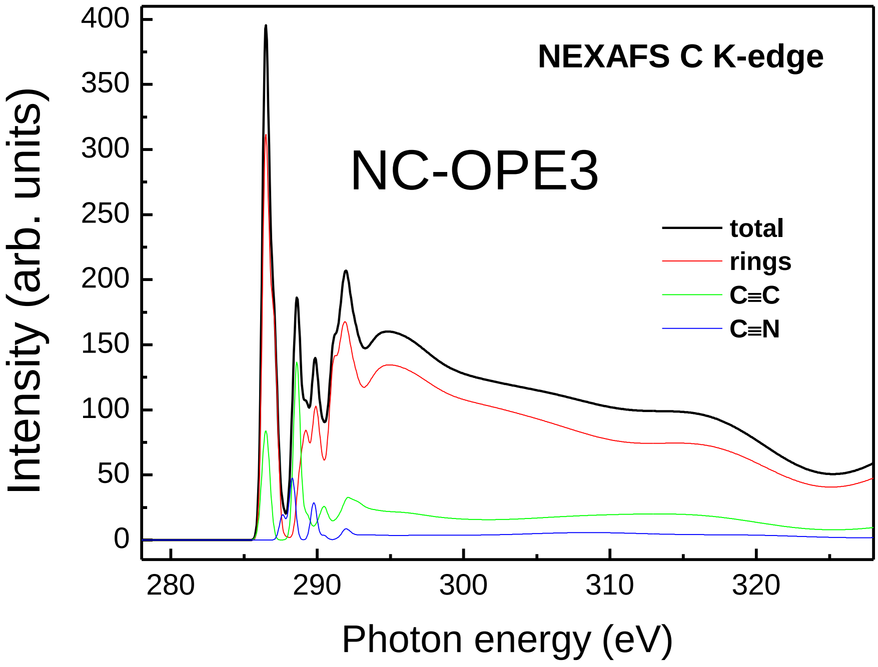

Figure S3: Calculated C K-edge NEXAFS spectra of NC-OPE3 in the twisted conformations. The spectrum is decomposed into the contributions related to the individual functional units.

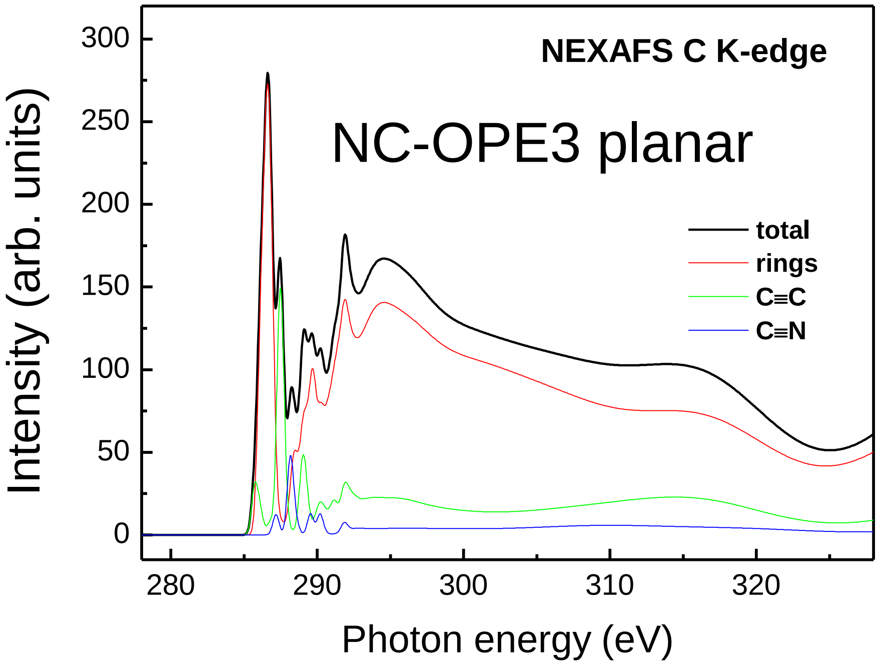

Figure S4: Calculated C K-edge NEXAFS spectra of NC-OPE3 in the planar conformations. The spectrum is decomposed into the contributions related to the individual functional units.

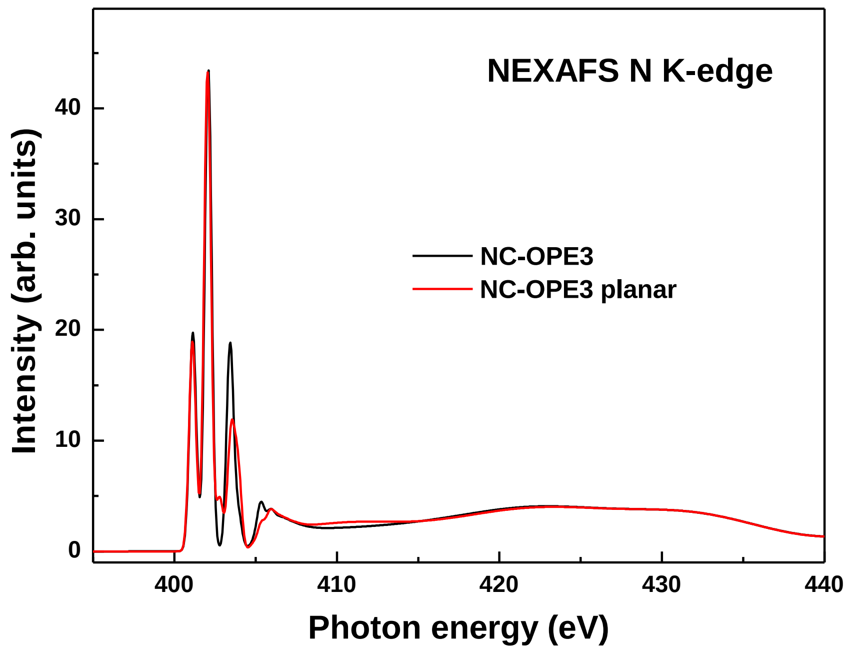

Figure S5: Calculated N K-edge NEXAFS spectra of NC-OPE3 in the planar and twisted conformations.
